# Analysis of the key component puff-by-puff transport in special segments and aerosol for electrical heated tobacco product

**DOI:** 10.3389/fchem.2024.1477795

**Published:** 2024-11-18

**Authors:** Xinyan Jin, Decai Meng, Lili Fu, Yang Zhao, Qi Zhang, Zhan Zhang, Xianzhong Yin, Qinpeng Shen, Shuang Wang, Yue Zhang, Le Wang, Ping Lei, Bin Li

**Affiliations:** ^1^ Zhengzhou Tobacco Research Institute of CNTC, Zhengzhou, Henan, China; ^2^ Technical Center of Yunnan China Tobacco Industry Corporation, Kunming, Yunnan, China; ^3^ China Tobacco Henan Industrial Co., Ltd., Zhengzhou, China

**Keywords:** heated tobacco products, puff-by-puff release, analytical model, closed-ended, nicotine

## Abstract

Understanding the puff-by-puff delivery mechanisms of key components of heated tobacco products is critical to developing product designs. This study investigates the puff-by-puff release patterns of key components in Natural Smoke Cigarettes (NSCs), which are designed to deliver nicotine without combustion by reducing oxygen content, utilizing a 30-s puff interval, a 2-s puff duration, and a 55 mL puff volume to simulate realistic smoking conditions. By establishing models to analyze the variation of nicotine, glycerol, 1,2-propylene glycol (PG), and water in different functional sections of the cigarette under controlled smoking conditions. These sections include the tobacco, hollow, cooling, and filter sections, constituting the structure of NSCs. In addition, the model calculates the port-by-port release of the components in the flue gas aerosol and compares it with the measured value. The results showed that: The retention amount in the tobacco section showed a steep decline in the first three puffs, with an overall exponential decrease. The amounts in the other sections were consistent, increasing in the first two puffs and then stabilizing. The retention amount decreased linearly with each puff, with a similar pattern across sections. The release amount peaked at the fourth to fifth puffs and then stabilized. The retention amount in the tobacco section declined exponentially in the first three puffs. It peaked in the second to third puffs in other sections, then decreased with each subsequent puff. The retention amount in the tobacco section showed a significant decline in the first puff, stabilizing at around 4 mg. In other sections, it peaked at the first puff and then rapidly declined. These findings can inform the development of reduced-harm smoking products and contribute to a better understanding of the dynamics of smoke generation. Additionally, the study offers a reference for the puff-by-puff release stability of NSCs and the improvement of consumers’ sensory quality.

## 1 Introduction

The health risks associated with smoking have garnered global attention (Baker, 2006). Under normal circumstances, the puffing temperature of traditional cigarettes can be as high as 900°C (Zhou et al., 2011). Under high-temperature conditions, tobacco leaves or materials undergo combustion, pyrolysis, and thermal synthesis (Baker, 2006), generating many potentially harmful substances (Harmful and Potentially Harmful Constituents, HPHCs). Traditional cigarettes are known to contain over 50 carcinogens (Postma et al., 2015), including polynuclear aromatic hydrocarbons (PAHs), aromatic amines, and nitrosamines.

As consumers increasingly prioritize health and global tobacco control efforts intensify, tobacco manufacturers have shifted their focus toward developing and promoting electronic nicotine delivery systems (ENDS), heated tobacco products (HTPs), and oral nicotine products (ONPs) as alternatives to traditional cigarettes. This shift aims to reduce the release of harmful substances and minimize the adverse health effects associated with smoking (Tattan-Birch et al., 2022). ENDS is an electronic product that mimics a cigarette and has a similar appearance, smoke, taste, and feel as a cigarette. It uses atomization and other means to turn the nicotine solution into a vapor for the user to smoke. E-cigarettes do not contain tobacco but use liquids containing nicotine, flavors, and other chemicals. ONPs: These products do not involve a combustion process and do not produce smoke. They include forms such as mouth smoke, snuff, and chewing tobacco. Smoke-free tobacco products are often used to transition to smoking cessation or to reduce the risk of inhaling harmful substances by providing consumers with a sense of satisfaction through the ingestion, inhalation, and chewing of tobacco products. Heated tobacco products are novel tobacco items that heat the tobacco (at or below 350°C) without combustion (Borgerding et al., 1998), thereby significantly reducing the emission of harmful constituents (Pryor et al., 1990; deBethizy et al., 2013) while providing consumers with a similar tobacco experience. Compared to conventional cigarettes, the harmful substances in the aerosol of IQOSTM products can reduce emissions by more than 90 percent compared to these toxic substances measured in the mainstream smoke of 3R4F cigarettes (Niu et al., 2023; Tabuchi et al., 2018). According to CORESTA (CORESTA Recommended Method No. 99, 2023; CORESTA Recommended Method No. 100, 2023; CORESTA Recommended Method No. 101, 2023; CORESTA Guideline No. 11, 2020; CORESTA Guideline No. 12, 2020) (Cooperation Centre for Scientific Research Relative to Tobacco) and ISO (ISO 10185, 2004; ISO 6488, 2004; ISO 13586, 2017) (International Organization for Standardization), heated tobacco products (HTPs) are categorized into three types: electrically heated tobacco products (eHTPs), carbon heated tobacco products (cHTPs), and aerosol heated tobacco products (aHTPs). The eHTPs include Philip Morris International’s iQOS and British American Tobacco’s Glo, while the cHTPs are represented by Reynolds’ REVO, and the aHTPs by Japan Tobacco’s Ploom TECH and KT&G’s Lil from Korea. Table 1 shows us the characteristics of the different systems.

**TABLE 1:** Characteristics of different tobacco products.

Aerosol heated tobacco products (aHTPs)	Electrical heated tobacco products (eHTPs)	Carbon heated tobacco products (cHTPs)
Characteristics		
Aerosol heating/carrier bands and retention	Electric heater + cigarettes	There is no need for a matching set to heat the cigarette
The consumption cost is slightly higher	Heat is supplied through an electric heater	Heating tobacco without burning it produces a vapor containing nicotine
There are no special mature products	Commercialization was successfully realized and market competition intensified	Tobacco + carbonaceous heat source

Currently, heated tobacco products are in a period of intense product and technology development (Simonavicius et al., 2019; Kim, 2018; Li et al., 2019). Heated tobacco products can be divided into “Heated not burn (HNB)” and “Natural Smoke Cigarettes (NSC).” The fundamental difference between the two is that the airflow path is different. According to whether the cold air flows through the tobacco section, the former is “Open-Ended” and the latter is “Closed-Ended.” HNB promotes the transfer and cooling of high-temperature aerosol precursors through cold air flowing through the tobacco segment to form aerosols. Compared to other heated tobacco products, Natural Smoke Cigarettes (NSC) feature a sealed base and perforated sidewalls in the hollow segment. During smoking, air does not flow through the tobacco section, but enters through these perforations, creating negative pressure, while the aerosol generated by heating the tobacco section creates positive pressure. This pressure difference facilitates the transfer of aerosol smoke. As the air does not pass through the tobacco section, the resulting aerosol has a lower temperature and higher transfer efficiency, garnering significant attention within the industry (Xudong et al., 2018). Zhengzhou Tobacco Research Institute and China Tobacco Yunnan jointly proposed and constructed the new concept of NSC and cigarette products category. Previous studies by Li et al. (2022) compared NSC with conventional heated tobacco products, revealing that under the same heating conditions, NSC exhibited higher aerosol collected mass (ACM) and transfer rates for nicotine, glycerol, and PG. Current research on heated tobacco products primarily focuses on toxicological evaluation, aerosol particle size and concentration distribution, chemical composition of the aerosol, and the effects of heating temperature (Xudong et al., 2018), puffing patterns (Hongfei et al., 2018), and tobacco materials on aerosol generation and release. For instance, the carcinogen content in the smoke of Eclipse (tobacco products developed by Reynolds) was 80% lower than that of regular cigarettes, reducing smokers’ intake of mutagens by 70%, and lowering the incidence rates of bronchitis and pneumonia by 46% and 36%, respectively (Foy et al., 2004; ECLIPSE Expert Panel, 2000). This significantly mitigates the health risks associated with tobacco products. Zhang et al. (2024) utilized a vacuum ultraviolet photoionization time-of-flight mass spectrometer (PI-TOFMS) for real-time detection and quantitative analysis of aerosol components in both conventional heated tobacco products and NSC. Zhongya et al. (2022) explored the impact of oxygen concentration on the chemical components of aerosols under low-temperature heating from a thermal transformation perspective, using tobacco raw materials as the research subject. Mengqi et al. (2022) studied the distribution of smoke chemical components in different parts of heated tobacco products under various puffing modes. Nordlund and Kuczaj (2016) utilized a numerical model based on extended Classical Nucleation Theory (CNT) for multicomponent gas mixtures to simulate the nucleation process in the Electrically Heated Tobacco System, finding that aerosol droplets form only in the presence of an aerosol former, primarily glycerol. Lulu et al. (2023a) investigated the impact of airflow channel design on the puff-by-puff smoke components of NSC, examining how different perforation parameters affect the puff-by-puff release of key components in mainstream smoke, the retention rates of components in different sections of a cigarette after smoking, the average smoke exit temperature, and average draw resistance.

Researchers such as Guo et al. (2018) have utilized thermogravimetric analysis (TG) and online nuclear magnetic resonance analysis (NMR) to study the drying kinetics and determine the effective moisture diffusion coefficient of fresh tobacco leaves during the drying process. To quantify the relationship between the pyrolysis characteristics and chemical composition of tobacco raw materials, Wu et al. (2024) and colleagues developed a quantitative analysis model using various machine learning methods to establish a link between tobacco weight loss rate and 19 chemical components. However, the release kinetics of tobacco leaves and cut tobacco differ from those within cigarettes. There is a lack of research on the variations in key substance content in different sections of NSC, the release and filter retention mechanisms, and the aerosol release dynamics.

This study examines the puff-by-puff variation of key components (nicotine, glycerol, PG, and moisture) in all segments of NSC (tobacco section, hollow section, cooling section, and filter section), with puffing intervals of 30 s and a puff volume of 55 mL. By analyzing the puff-by-puff variation of key components in different functional segments of NSC, and calculating the puff-by-puff release quantities of these components based on mass balance relationships, the study aims to investigate the release mechanism of NSC smoke and regulate the puff-by-puff release quantities. Our study aims to provide a reference for the puff-by-puff release stability of natural smoke cigarettes and enhance the sensory quality for consumers, which will ultimately assist in optimizing the design of heated tobacco products.

## 2 Materials and methods

### 2.1 Experimental materials

**FIGURE 1:**
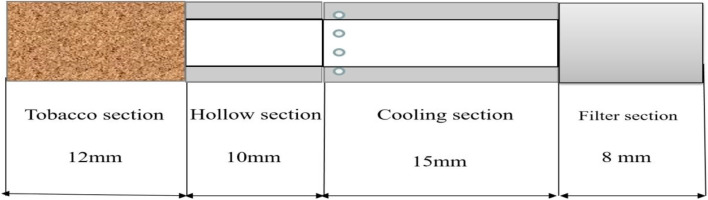
Schematic diagram of heated tobacco products.

The heated tobacco products used in this experiment were Natural Smoke Cigarette samples provided by China Tobacco Henan Industrial Co., LTD. NSC: the total length of the cigarette is 45 mm, the circumference is 22.5 mm, the length of the tobacco section is 12 mm, the length of the hollow section is 10 mm, the length of the cooling section is 15 mm, and the length of the filter section is 8 mm. A schematic diagram of cigarettes is shown in Figure 1. The hollow section is made of paper material, and the cooling section and filter section are made of acetate fiber material. Cigarettes weigh about (510 mg ± 10 mg). The initial content of water in the tobacco section is about 15 mg, the initial content of PG is about 1.1 mg, the initial content of nicotine is about 1.3 mg, and the initial content of glycerol is about 20 mg. The cigarettes were stored at room temperature before the experiment.

**FIGURE 2:**
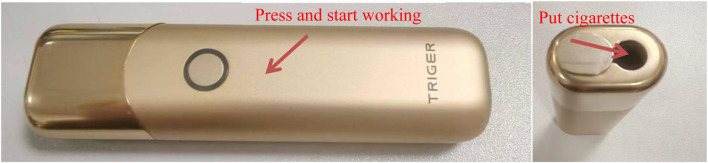
A physical picture of a cigarette set.

**FIGURE 3:**
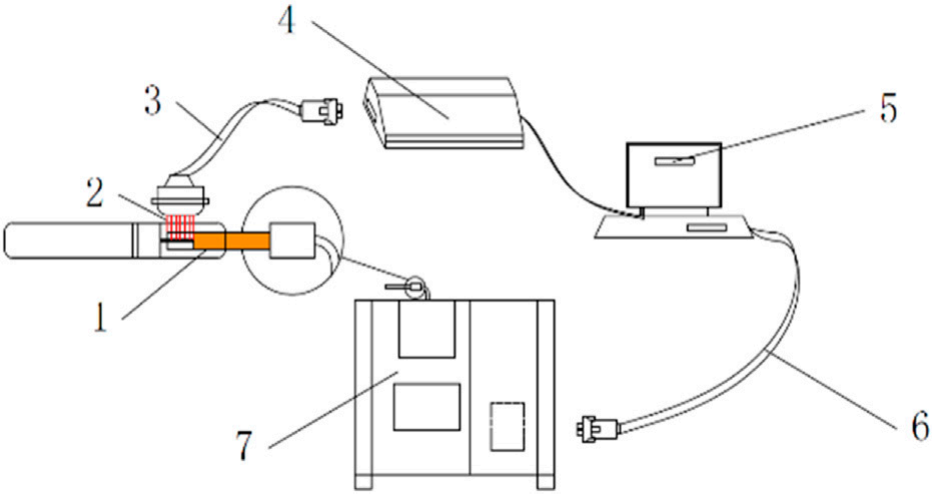
Schematic diagram of the device for cigarette temperature detection (Wang et al., 2021). 1 Heating cigarette to be tested. 2 Thermocouple module. 3 Thermocouple compensation wire. 4 Data collector. 5 DAQ Data Acquisition software. 6 Data Connection cable. 7 Temperature-controlled single-channel smoking machine.

Heated-tobacco device: The device is a circumferential heating type. The manufacturer of the device is Shenzhen Huayu Technology Development Co., Ltd. and the maximum output is 40W. The working time of the device is 3 min. The physical picture of the cigarette set is shown in Figure 2. The cigarette temperature detection device shown in Figure 3 is used to detect the temperature of the cigarette set and cigarette stand, including the heated cigarette to be tested, the thermocouple module, the thermocouple compensation line, the data collector, the DAQ data acquisition software, the data connection line, and the temperature-controlled single-channel smoking machine. We employed a thermocouple to measure the operating temperature of the smoking device (the temperature at the inner surface of the heating tube of the device) and the center temperature of the cigarette during puffing. To measure the operating temperature of the device, the thermocouple was inserted at the interface where the cigarette comes into contact with the device. To measure the center temperature of the cigarette, the thermocouple was inserted into the center of the cigarette.

#### 2.1.1 Reagents

We utilized the following reagents in our study: N-heptadecane (≥99.5%, sourced from Aladdin, United States), nicotine (chromatography pure, obtained from Sigma, United States), 1,2-propylene glycol (≥99.5%, purchased from Sigma-Aldrich, United States), glycerol (≥99.0%, sourced from Sigma-Aldrich Corporation), heptadecane (99%, purchased from Sigma-Aldrich Corporation, United States), 1,4-butanediol (99%, purchased from Sigma-Aldrich Corporation, United States), and methanol (chromatographic pure, sourced from Shanghai Aladdin Biochemical Technology Co., LTD.).

#### 2.1.2 Machine learning methods

**FIGURE 4:**
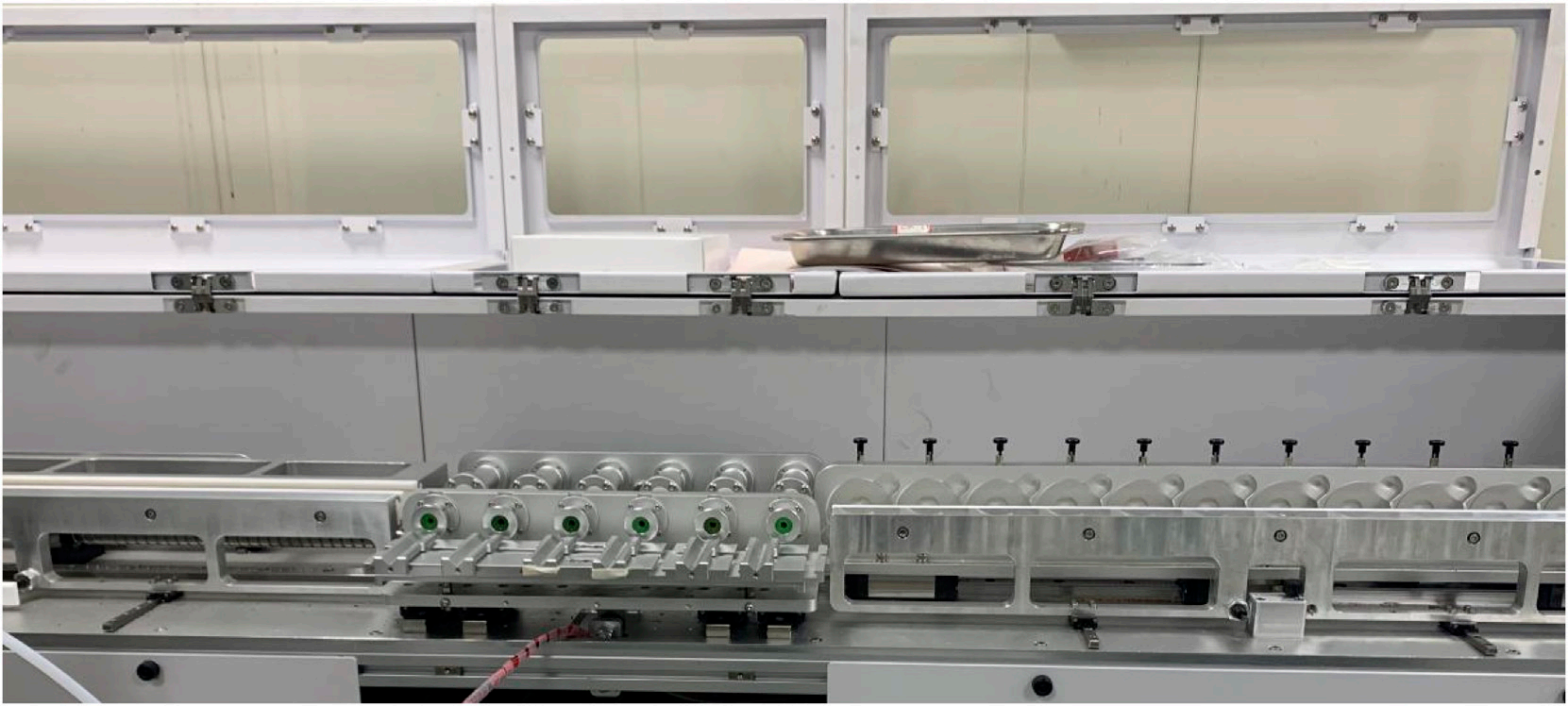
i‒MAC600A array type multi-port puff-by-puff smoking machine.

i‒MAC600A array type multi-port puff-by-puff smoking machine (manufactured by Zhengzhou tobacco research institution of CNTC (Lulu et al., 2023b)), the physical picture of the smoking machine is shown in Figure 4. The smoking machine primarily consists of a puffing module, a trap feed module, and a clamping and sealing module. Different puffing curves and capacities can be achieved by adjusting the motor speed and piston stroke in the puffing module. The use of multi-channel simultaneous puffing enhances detection efficiency. Sequential control technology is employed to switch the trap puff-by-puff, enabling the collection of aerosol on a puff-by-puff basis.

We utilized the following instrumentation in our study: a 7,890 Gas Chromatograph equipped with FID and TCD detectors, sourced from Agilent, United States; a CP2245 electronic balance with a sensitivity of 0.0001 g, manufactured by Sartorius, Germany; and a Hy-8 speed regulating Oscillator, produced by Changzhou Guohua Electric Appliance Co., LTD.

### 2.2 Methods

#### 2.2.1 Determination of water, PG, glycerol, and nicotine

The detection methods for water, PG, glycerol, and nicotine in tobacco samples from the tobacco section, hollow section, cooling section, filter section, and aerosol were based on the procedures outlined by Kensengye et al. (Shengye et al., 2020), Wang Kang et al. (Kang et al., 2019), and the tobacco industry standard YC/T154-2001 (State Tobacco Monopoly Administration, 2001). The specific experimental procedure is as follows:

Sample Preparation: The smoked cigarettes were cut open with a blade to obtain the various functional sections, including the filter section, tobacco section, hollow section, and cooling section.

Extraction Process: Each functional section was placed separately into a 50 mL centrifuge tube. For example, six filter sections were placed in one 50 mL centrifuge tube. The corresponding volume of extraction solvent was then added (35 mL for the tobacco section and 25 mL for the filter, hollow, and cooling sections). The extraction solvent was a methanol solution containing 0.2 mg/mL n-heptadecane. The samples were shaken for 3 h and left to stand overnight. For the blank sample, 25 mL of extraction solvent was added to a 50 mL centrifuge tube, shaken for 3 h, and left overnight.

Analysis Process: After extraction, the extract was used for further analysis. The overnight-treated samples were filtered into chromatography vials using syringes and filters. Gas chromatography was then performed to determine the content of key components in each functional section.

#### 2.2.2 Chromatographic conditions

An HP-INNOWAX capillary column (30 m × 0.32 mm × 0.25 μm) was used for chromatography. The heating procedure involved a temperature ramp from 60°C to 250°C at a rate of 20°C/min with an analysis time of 15 min. The inlet temperature was set at 250°C, the TCD detector temperature at 260°C, and helium (>99.999%) was used as the carrier gas at a flow rate of 6 mL/min in constant flow mode. Additionally, tail-blowing utilized helium gas at a flow rate of 9 mL/min. The injection volume was maintained at 1 μL, employing a shunt injection mode with a split ratio of 5:1.

**FIGURE 5:**
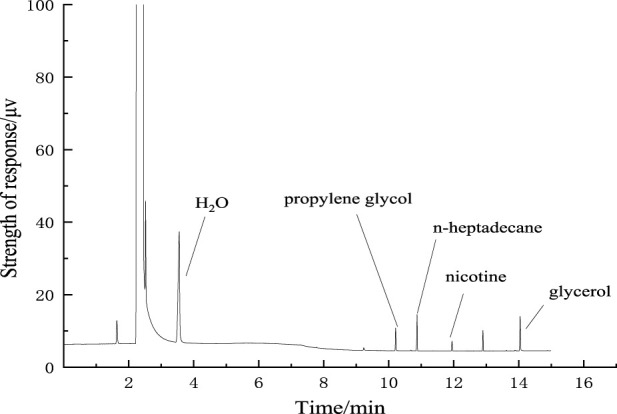
Chromatogram of separation of key components.

Water, propylene glycol, nicotine, and glycerol were completely separated from the internal standard n-heptadecane, and the chromatographic peak shape was sharp and without tail, as shown in Figure 5.

**FIGURE 6:**
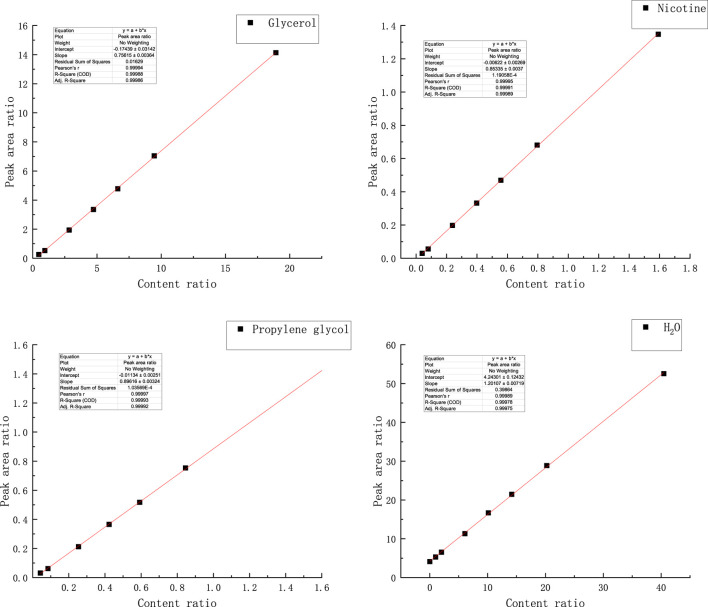
Standard curves of each key component on gas chromatography.

The standard curves of water, propylene glycol, nicotine, and glycerol were established by using the internal standard method, as shown in Figure 6, and then the accurate quantification of each key component was realized: the peak area ratio of water to the internal standard and the content ratio of water to internal standard showed a significant linear relationship between the coefficient of determination R^2^ of 0.99975. The standard curve R2 of propylene glycol reached 0.99992. Nicotine standard curve R^2^ reached 0.99989; The R^2^ of the glycerol standard curve reached 0.99986.

#### 2.2.3 Puffing parameters

Health Canada Puffing model was performed here, (bell curve puffing, puffing volume was 55 mL, puffing duration was 2 s, puffing interval was 30 s), and all the experimental cigarettes were smoked for 7 puffs.

#### 2.2.4 Sample extraction grouping

Samples are grouped and tested according to tobacco industry standard YC/T345-2010 (State Tobacco Monopoly Administration, 2010). In addition, the number of extracted cigarettes after puffing was determined by the variation in release amounts from each port. The quantity of extracted cigarettes varied according to the number of smoked ports: for the first and second puffs, there were 6 parallel groups in the filter section, 6 parallel groups in the cooling section, 3 parallel groups in the hollow section, and 2 parallel groups in the cut tobacco section. The third and fourth puffs were similar, with 3 and 4 parallel groups in the filter, cooling, and hollow sections, respectively, while the tobacco section had 4 and 4 parallel groups. For the fifth puff, there were 3 parallel groups in the filter section, cooling section, and hollow section, with the tobacco section having 5 parallel groups of three. The sixth puff had two sets of six for the filter section, cooling section, and hollow section, with seven sets for the tobacco sections. The seventh puff maintained two sets of six for the filter and cooling sections and featured eight sets with two parallel groups for the tobacco sections.

#### 2.2.5 Model construction and optimization

In the present study, utilizing the least squares method within the Origin data analysis software, linear or exponential fits were performed on the data about the key components of each cigarette segment. The least squares method was selected for its ability to minimize the sum of the squares of the residuals, thus ensuring that the models accurately represent the underlying data patterns.

For each segment of the cigarette, the changes in the concentrations of the key components were analyzed, and by aggregating these changes, the emission profile of each component in the flue gas was deduced.

To evaluate the accuracy of the models and to optimize the predictions, Mean Absolute Error (MAE) and Root Mean Square Error (RMSE) were introduced as metrics. The formulas are Equations 1, 2, respectively. These statistical evaluations provided a quantitative assessment of the model’s predictive performance by measuring the average magnitude of the errors and the standard deviation of the residuals, respectively.

$$\text{MAE }\left(\text{Mean Absolute Error}\right):MAE=\frac{1}{n}\sum_{i=1}^{n}\left|y_{i}-\hat{y_{i}}\right|$$ (1)

$$R\text{MSE }\left(\text{Root Mean Square Error}\right):RMSE=\sqrt{\frac{1}{m}{\sum_{i=1}^{m}\left(y_{i}-\hat{y_{i}}\right)}^{2}}$$ (2)

$y_{i}$ is the experimental value; $\hat{y_{i}}$ is the revised calculated value.

## 3 Results and discussion

**FIGURE 7:**
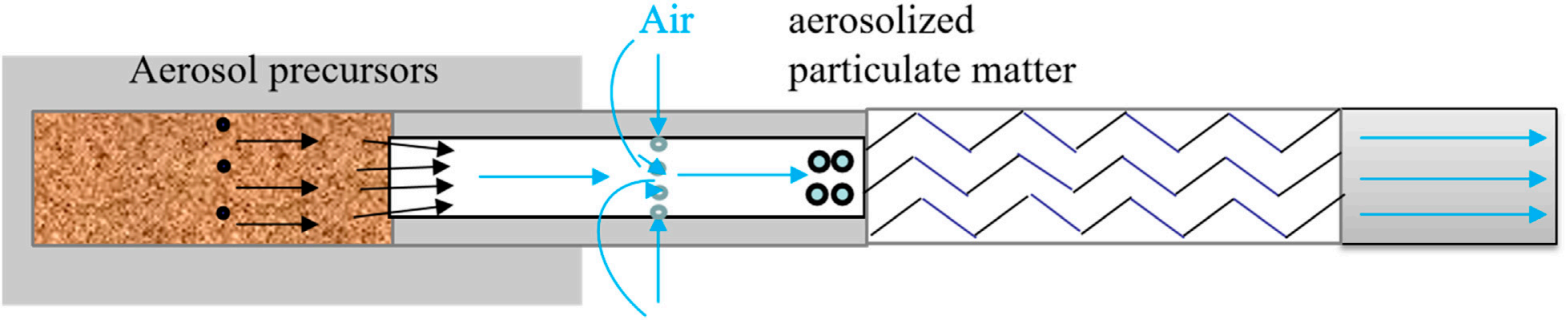
Generation and delivery of key components.

Analytical models were developed to examine the sequential changes in critical components of aerosol from natural tobacco cigarettes. These models were established to analyze the evolving patterns of these components across the tobacco section, hollow section, cooling section, and filter section. Mass balance calculations were employed to determine the quantity of each key component released in aerosol, which was then compared against measured values obtained from sequential aerosol release detection. The generation and delivery of key components during vaping is shown in Figure 7. Natural smoke cigarettes, air from the side wall openings into the role of the pressure difference between the aerosol precursors and air in the hollow section of the encounter cooling, nucleation, and ultimately the formation of aerosols.

### 3.1 Operating temperature curve of smoker

**FIGURE 8:**
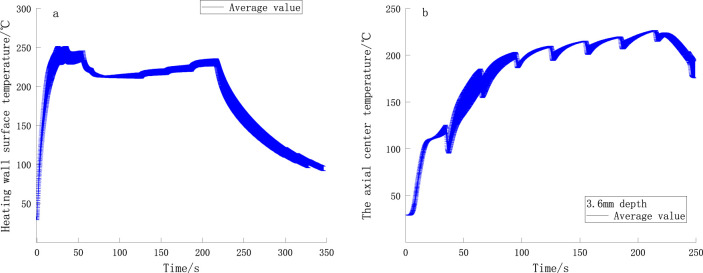
**(A)** Temperature curves of the heating wall and **(B)** Temperature curves of axial center of the smoker.

The temperature profile of the heated wall is shown in Figure 8A, and the axial center temperature profile of the heating device is shown in Figure 8B. It can be observed that the temperature deviations in multiple measurements of the inner surface of the heating tube and the axial center of the device are minimal, indicating that the operating temperature of the device is relatively stable. The initial temperature of the device is approximately 22°C. After heating begins, the wall surface temperature rises rapidly (within 0–25 s), reaching 250°C at 50 s before decreasing to 220°C. As time progresses, the wall surface temperature increases in a stepwise manner. The temperature at the axial center of the device (3.6 mm depth) naturally increases with the rise in wall surface temperature, stabilizing at approximately 220°C.

### 3.2 Calculation of cumulative values for key components

To validate data reliability, cumulative amounts of nicotine, glycerol, PG, and moisture content in sequential smoke were calculated and compared against their respective initial values. The calculation method is as follows:

#### 3.2.1 Key components of the port accounting calculation method

Initial value of each key component:

$$m_{0}=m_{\text{core}\;}+m_{\text{hollow}}+m_{\text{cooling}}+m_{\text{filter}}$$ (3)

Total value of key components per mouthful:

$$m_{n,i}=m_{\text{core}}+m_{\text{hollow}}+m_{\text{cooling}}+\sum_{i=1}^{n}m_{\text{aerosol}}$$ (4)

here, n represents the number of puffs, n < 8. i=(1, 2, 3, 4); 1 represents the tobacco section; 2 represents the hollow section; 3 represents the cooling section; 4 represents the filter section.

**FIGURE 9:**
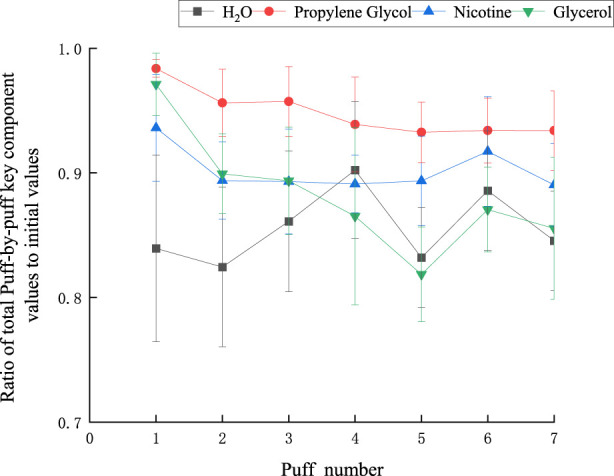
The ratio of the total value of each key component to the initial value at each port sequence.

With a puff interval of 30 s and a puff volume of 55 mL, the ratio of cumulative values to initial values for each key component is illustrated in Figure 9. Notably, the ratio for nicotine remains consistently above 0.8, while that of PG consistently exceeds 0.9. The moisture content exhibited significantly greater fluctuations, with values exceeding 0.7, compared to other components. This is attributed to the initially higher moisture levels and its susceptibility to environmental influences. The overall trend of mass loss increased with the number of puffs. Among the first five puffs, the conservation ratio of glycerol showed the most evident decline, likely because the boiling point of glycerol (290°C) is higher than that of other components, making it more prone to condensation on the device, thus leading to mass loss.

These offer a reference for forecasting aerosol emission: Generally, the cumulative value of each component per mouthful fails to achieve mass conservation (that is, the cumulative value per mouthful divided by the initial value is not equal to 1). It indicates that there is a certain amount of quality loss during the experimental test. During the experiment, a portion of the smoke will condense and adhere to the inner wall of the smoker. This process not only causes a reduction in the total amount of aerosol but, more significantly, leads to the deposition of key components in the aerosol. This implies that even if theoretically the sum of the changes in the components of each section of the cigarette can reflect the total amount of components in the aerosol in practice, due to condensation, this part of the component does not fully enter the collector, resulting in actual measurement values being lower than expected. When the average cumulative value per puff is λ of the initial value, it indicates that the efficiency with which the actual key components are captured is λ. So the correction coefficient value of the model for forecasting aerosol emission is λ.

### 3.3 Nicotine release analysis model

**FIGURE 10:**
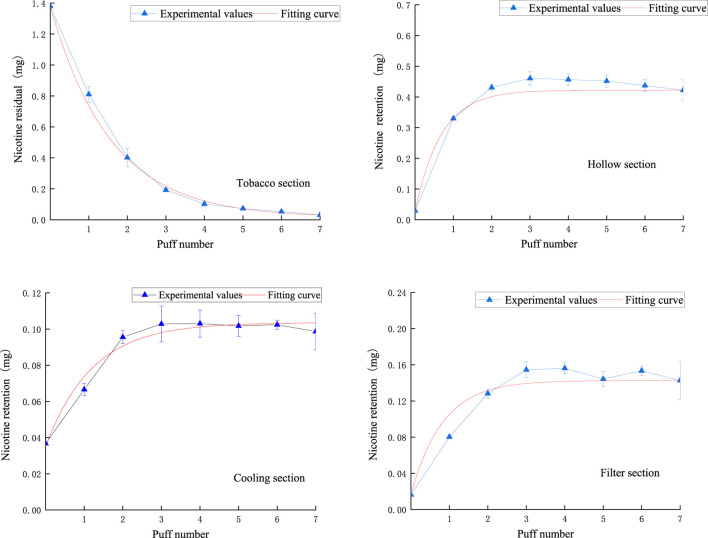
The retention amount of nicotine in the tobacco section and the amount of nicotine trapped in the hollow section, cooling section, and filter section.

The results of comparing the fitted curves and experimental values of nicotine in the core, hollow, cooling, and filter sections are shown in Figure 10. It can be observed that the fitted curves closely match the experimental results, demonstrating a strong regularity. Specifically, the remaining nicotine in the tobacco section puff-by-puff follows an exponential function relationship, with a rapid decrease in the first three puffs followed by a more gradual decline. This leads to the instability of nicotine release in the aerosol. The stable release of nicotine in the aerosol is crucial for the sensory quality of NSC, so in order to improve the stability of product release, the first step is to control the stable release of nicotine in the tobacco section.

In the hollow section, the nicotine content puff-by-puff also follows an exponential function relationship, gradually increasing in the first three puffs and remaining stable after reaching an adsorption equilibrium, then slightly decreasing as the number of puffs increases. The cooling section’s nicotine content puff-by-puff follows an exponential function relationship, with a gradual increase in the first three puffs, reaching adsorption equilibrium, and then remaining stable. Similarly, in the filter section, the nicotine content puff-by-puff follows an exponential function relationship, increasing in the first three puffs and stabilizing after reaching adsorption equilibrium. This suggests that after the third puff, the adsorption of nicotine in the hollow section, cooling section, and filter section approaches saturation. Meanwhile, by comparing the order of magnitude in the figure, it can be seen that the retention of nicotine is highest in the hollow section and lowest in the cooling section. This may be due to the fact that the material in the cooling section has a stronger adsorption capacity for nicotine. Therefore, the material of the hollow section of the NSC can be replaced with the same material of the cooling section in the product design.

The analysis model for nicotine in various functional sections of natural smoke cigarettes, as calculated from Figure 7, is as follows:

Nicotine remaining in the tobacco section:

$$m_{n,1}{\left(n\right)=0.0153+1.3641e}^{-0.6374n};\;R^{2}=0.99578$$ (5)

Nicotine retention in the hollow section:

$$m_{n,2}{\left(n\right)=0.4220+0.3292e}^{-1.4613n};\;R^{2}=0.98585$$ (6)

Nicotine retention in the cooling section:

$$m_{n,3}{\left(n\right)=0.1036-0.06862e}^{-0.8335n};\;R^{2}=0.95571$$ (7)

Nicotine retention in the filter section:

$$m_{n,4}{\left(n\right)=0.1427-0.1263e}^{-1.2161n};\;R^{2}=0.93768$$ (8)

The puff-by-puff changes in nicotine content in each functional section can be obtained by calculating the differential values:

$$\Delta m_{n,i}\left(n\right)=m_{n,i}\left(n\right)-m_{n,i}\left(n-1\right)$$ (9)

here, n represents the number of puffs, n < 8.

**FIGURE 11:**
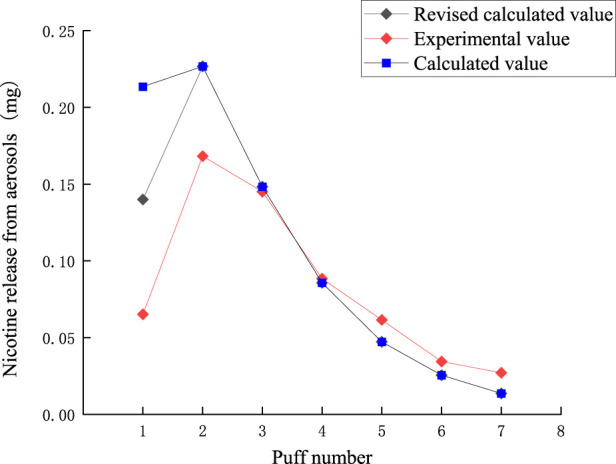
Comparison of calculated and experimental values of nicotine release in aerosol.

The puff-by-puff release of nicotine from the aerosol can be calculated by summarizing the data from the puff-by-puff changes in each functional segment. The comparison between the calculated and experimental values of nicotine release in the aerosol is shown in Figure 11. There is a certain deviation between the experimental and calculated values in the first two puffs. Due to the loss of nicotine adsorbed onto the smoking apparatus and other dissipation, the nicotine content cannot be fully detected. Referring to 3.2, it can be seen that the average actual capture efficiency of nicotine per port is 0.9. Therefore, a correction factor λ = 0.9 was introduced into the model for predicting aerosol release. The RMSE value between the predicted results and the measured nicotine values was 0.037, the MAE value is 0.025. The results demonstrated a significant reduction in the deviation between the corrected and experimental values for the first puff, with a relative deviation of approximately 0.08 mg. For the third to seventh puffs, the relative deviation was about 0.01 mg. Thereby, the accuracy of the prediction of nicotine release per puff was improved. Highly accurate predictive modeling can help us better control the release of nicotine, thereby improving the sensory experience and safety of our products. Nicotine puff-by-puff release:

$$q_{n}\left(n\right)=\lambda \left(n\right)\Delta m_{n,1}\left(n\right)+\Delta m_{n,2}\left(n\right)+\Delta m_{n,3}\left(n\right)+\Delta m_{n,4}\left(n\right)$$ (10)

### 3.4 Glycerol release analysis model

**FIGURE 12:**
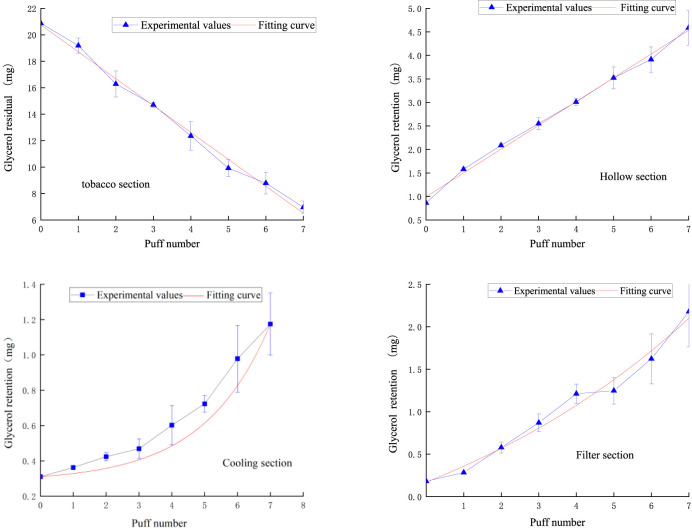
Retention amount of glycerol content in tobacco section, hollow section, cooling section, filter section.

Figure 12 demonstrates the fitted curves of glycerol content in each functional segment compared to the experimental values. In the tobacco segment, the glycerol residual tended to decrease linearly with the number of puffs. This phenomenon suggests that the release of glycerol within the tobacco segment is relatively uniform and continuous, probably due to the relatively uniform distribution of glycerol in tobacco or its relatively constant evaporation rate during the heating process. After entering the hollow segment, the glycerol content increased linearly puff-by-puff. In the cooling section, the change of glycerol content followed an exponential function relationship, which showed a slow increase in the first three puffs, followed by a rapid increase. This pattern of change may be related to the condensation of glycerol due to the decrease in temperature. The volatilization of glycerol slows down as the temperature decreases, but its concentration gradually increases as more glycerol passes from the preceding functional segments. The slow increase in the first three passes may be due to the fact that the glycerol has not yet condensed sufficiently in the initial stage, while the subsequent rapid rise reflects the cumulative effect of glycerol at low temperatures. The pattern of glycerol content in the filter section was similar to that of the cooling section, which also followed an exponential function.

The analysis model for glycerol in various functional sections of natural smoke cigarettes is as follows:

Glycerol remaining in the tobacco section:

$$m_{g,1}\left(n\right)=20.7517-2.0339n\;{;R}^{2}=0.99229$$ (11)

Glycerol retention in the hollow section:

$${\;m}_{g,2}\left(n\right)=0.99345+0.5059n\;{;R}^{2}=0.99487$$ (12)

Glycerol retention in the cooling section:

$${\;m}_{g,3}\left(n\right)=0.1791-0.1311e^{0.2921n}{\;;R}^{2}=0.99293$$ (13)

Glycerol retention in the filter section:

$${\;m}_{g,4}\left(n\right)=-1.3103+1.4804e^{0.1192n}{\;;R}^{2}=0.97413$$ (14)

The puff-by-puff changes in glycerol content in each functional section can be obtained by calculating the differential values:

$$\Delta m_{g,i}\left(n\right)=m_{g,i}\left(n\right)-m_{g,i}\left(n-1\right)$$ (15)

here, n represents the number of puffs, n < 8.

**FIGURE 13:**
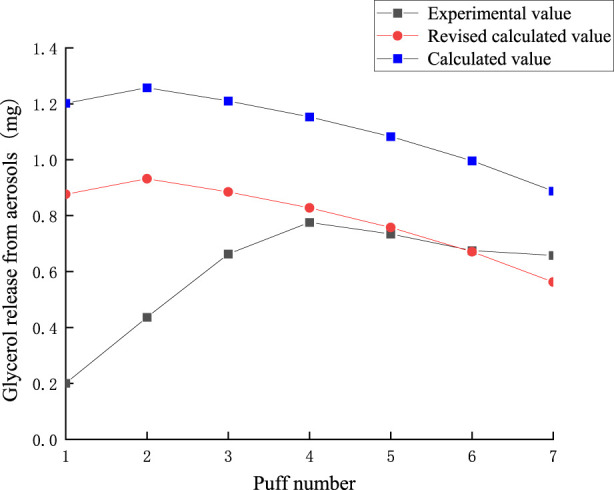
Comparison of calculated and experimental values of glycerol release in aerosol.

Summing the puff-by-puff changes across all sections provides the puff-by-puff release of glycerol in the aerosol. The comparison between the calculated and experimental values of glycerol release in the aerosol is shown in Figure 13. There is a significant deviation between the experimental and calculated values in the first three puffs. Due to the inability to fully detect glycerol content in heated tobacco products, the mass loss is substantial. Based on the transfer characteristics of glycerol in the tobacco segment and its relatively low transfer rate, the actual content of glycerol could not be fully detected considering the loss of glycerol during the transfer process and other dissipation factors. Referring to the results of the conservation of mass calculations in 3.2, it can be seen that the actual transfer efficiency of glycerol averaged per puff is 0.84. Therefore, in order to more accurately predict the aerosol release, a correction factor λ = 0.84 was introduced into the model. The RMSE value between the predicted results and the measured glycerol values was 0.33, the MAE value is 0.224. The results indicated that the deviation between the corrected and experimental values for each puff was significantly reduced, with the relative deviation for the fourth to seventh puffs being below 0.05 mg. The average relative deviation decreased, improving the accuracy of predicting glycerol release per puff. Glycerol puff-by-puff release:

$$q_{g}\left(n\right)=\lambda \left(n\right)\Delta m_{g,1}\left(n\right)+\Delta m_{g,2}\left(n\right)+\Delta m_{g,3}\left(n\right)+\Delta m_{g,4}\left(n\right)$$ (16)

### 3.5 PG release analysis model

**FIGURE 14:**
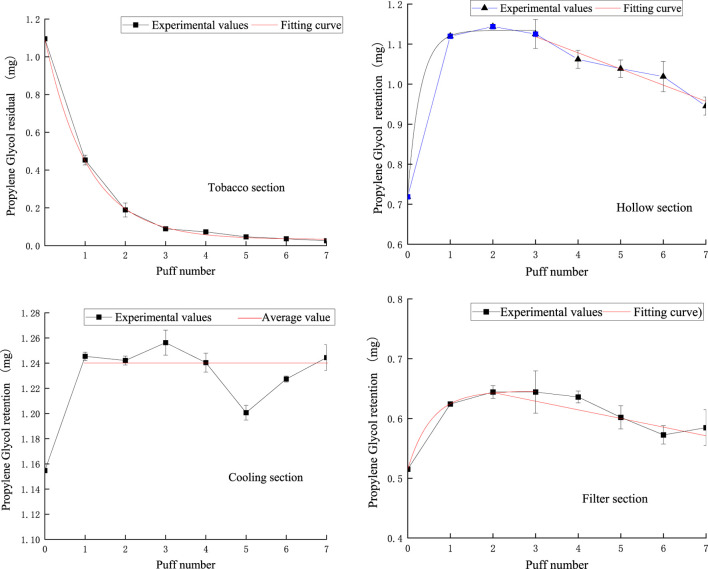
Retention amount of PG content in tobacco section, hollow section, cooling section, and filter section.

Figure 14 shows the puff-by-puff variation of propylene glycol content in each functional segment of NSC. The fitted curves were consistent with the experimental results and showed good agreement. Specifically, the amount of propylene glycol remaining in the tobacco segment decreased exponentially with the number of puffs, with a significant decrease in the first two puffs, after which the decrease gradually slowed down. This release pattern was similar to that of nicotine, suggesting that the release process of propylene glycol in the tobacco segment had similar dynamic characteristics.

In the hollow section, the content of propylene glycol showed an exponential change in the first three smoking cycles, and then gradually decreased after reaching a stable value in the first puff. This could be attributed to the large space provided by the hollow section, which allowed propylene glycol to be rapidly transferred from the tobacco section accumulated there, and then gradually released due to further suction. This phenomenon reveals the important role of the hollow section in the delivery and retention of propylene glycol.

The fluctuation of propylene glycol content in the cooling section from the first to the seventh pumping cycle was small, with a variation range of about ±0.02 mg, so we used 1.24 mg as the mean value of the fitted curve, reflecting the stability of this section in maintaining the propylene glycol content.

In the filter section, the propylene glycol content increased exponentially during the first three pumping cycles, but the content started to decrease from the third port. This change indicates that the filter nozzle section is not only involved in the retention of propylene glycol but also plays a role in its release. This dual role of the filter nozzle segment may be due to its internal structure and material properties, which are capable of both partial retention of propylene glycol and its gradual release in subsequent suction. This dynamic balance is essential to optimize the design of the filter nozzle for better taste and reduced release of harmful substances.

The analysis model for PG in various functional sections of natural smoke cigarettes is as follows:

PG remaining in the tobacco section:

$$m_{p,1}\left(n\right)=0.03285+1.06284e^{-0.9389n}{\;;R}^{2}=0.99936$$ (17)

PG retention in the hollow section:

$$m_{p,2}\left(n\right)=\left\{\begin{array}{l} \;1.1345-0.4157e^{-3.3914n},n\leq 3;R^{2}=0.9294\\ 1.\;2398-0.0404,n\geq 3;R^{2}=0.99572 \end{array}\right.$$ (18)

PG retention in the cooling section:

$$m_{p,3}\left(n\right)=\left\{\begin{array}{l} 1.155,n=0\\ 1.24,n\geq 1 \end{array}\right.$$ (19)

PG retention in the filter section:

$$m_{p,4}\left(n\right)=\left\{\begin{array}{l} 0.6460-0.1308e^{-1.8113n},n\leq 3;\;R^{2}=0.93768\\ 0.6724-0.01448n,n\geq 3;\;R^{2}=0.74592 \end{array}\right.$$ (20)

The puff-by-puff changes in PG content in each functional section can be obtained by calculating the differential values:

$$\Delta m_{p,i}\left(n\right)=m_{p,i}\left(n\right)-m_{p,i}\left(n-1\right)$$ (21)

here, n represents the number of puffs, n < 8.

**FIGURE 15:**
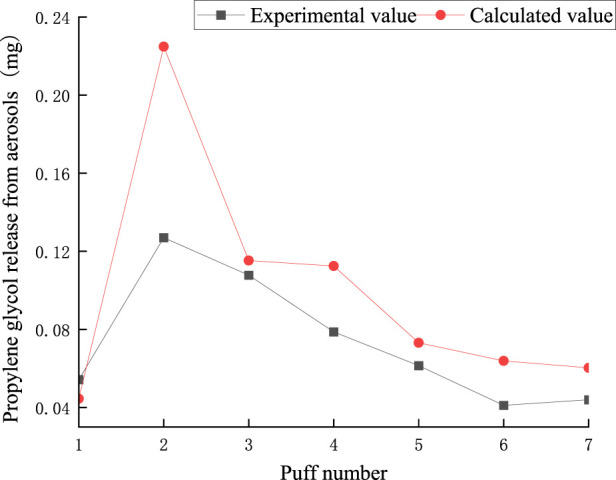
Comparison of calculated and experimental values of PG release in aerosol.

The puff-by-puff release of propylene glycol from the aerosol can be calculated by summarizing the data of the puff-by-puff changes in each functional segment. Figure 15 illustrates the calculated versus experimental values for the release of propylene glycol from aerosols. The RMSE between the predicted and measured propylene glycol values was 0.041, and the MAE was 0.029. There was a deviation of about 0.1 mg between the experimental and calculated values for the second puff. This is mainly due to adsorption losses of propylene glycol in e-cigarettes and other dissipative factors resulting in incomplete detection of its content. This bias could be reduced in the future by improving the initial start-up mechanism of the heating system or optimizing the material surface properties. However, the relative deviations for the other pumping cycles were less than 0.04 mg, suggesting that the model has a high accuracy in predicting the puff-by-puff release of propylene glycol. PG puff-by-puff release:

$$q_{p}\left(n\right)=\Delta m_{p,1}\left(n\right)+\Delta m_{p,2}\left(n\right)+\Delta m_{p,3}\left(n\right)+\Delta m_{p,4}\left(n\right)$$ (22)

### 3.6 Moisture release analysis model

**FIGURE 16:**
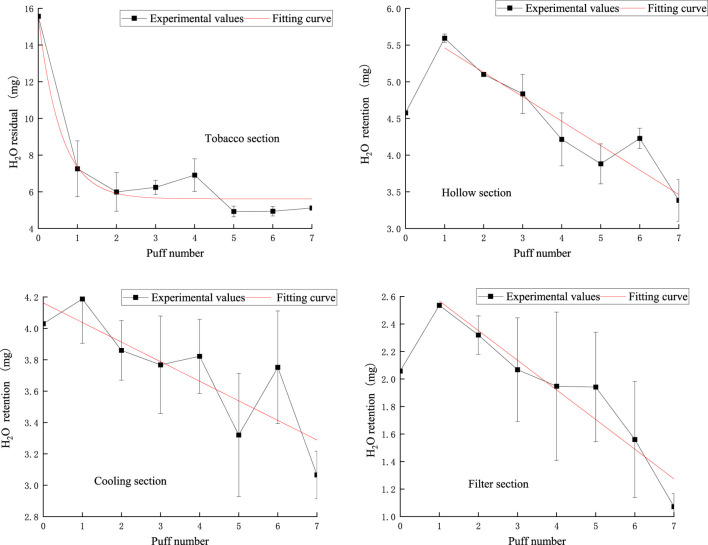
Retention amount of moisture content in the tobacco section, hollow section, cooling section, filter section.

The pattern of puff-by-puff moisture content in each functional section of the cigarette is shown in Figure 16.

In the tobacco section, the puff-by-puff moisture residual followed an exponential function relationship, with a sharp decrease in moisture at the first puff, followed by a gradual, flat decline. This rapid initial decrease may be due to the rapid evaporation of moisture at high temperatures.

In the hollow section, the puff-by-puff moisture content peaked at the first puff and then gradually decreased. This suggests that the hollow section absorbed and stored a certain amount of moisture in the initial stage, which was then gradually released with the pumping process.

The moisture content of the cooling section showed a linear decreasing trend. This linear decrease may be attributed to the lower temperature of the cooling section, which results in a slower but still continuous decrease in the rate of water evaporation. In addition, the large fluctuations and significant deviations in the cool-down section may be related to temperature control and material properties, which need to be further investigated to optimize the design.

The puff-by-puff moisture content of the filter section also decreased gradually after the first puff peaked. This suggests that the filter nozzle is not only capable of retaining moisture but also releasing it gradually in the subsequent suction process.

These phenomena suggest that the hollow section, the cooling section, and the filter mouthpiece section not only have the function of retaining moisture but also participate in the dynamic release of moisture. This finding provides an important reference for optimizing the design of tobacco products, especially in controlling moisture release.

The analysis model for moisture in various functional sections of natural smoke cigarettes is as follows:

Moisture remaining in the tobacco section:

$$m_{w,1}\left(n\right)=5.6101+9.9459e^{-1.7380n\;};\;R^{2}=0.9489$$ (23)

Moisture retention in the hollow section:

$$m_{w,2}\left(n\right)=\left\{\begin{array}{l} 4.5774,n=0\\ 5.7957-0.3332n,n\geq 1 \end{array}\right.;\;R^{2}=0.8832$$ (24)

Moisture retention in the cooling section:

$$m_{w,3}\left(n\right)=4.16254-0.1249n\;;\;R^{2}=0.65047$$ (25)

Moisture retention in the filter section:

$$m_{w,4}\left(n\right)=\left\{\begin{array}{l} 2.0579,n=0\\ 2.7833-0.2156n,n\geq 1 \end{array}\right.;\;R^{2}=0.90701$$ (26)

The puff-by-puff changes in moisture content in each functional section can be obtained by calculating the differential values:

$$\Delta m_{p,i}\left(n\right)=m_{p,i}\left(n\right)-m_{p,i}\left(n-1\right)$$ (27)

here, n represents the number of puffs, n < 8.

**FIGURE 17:**
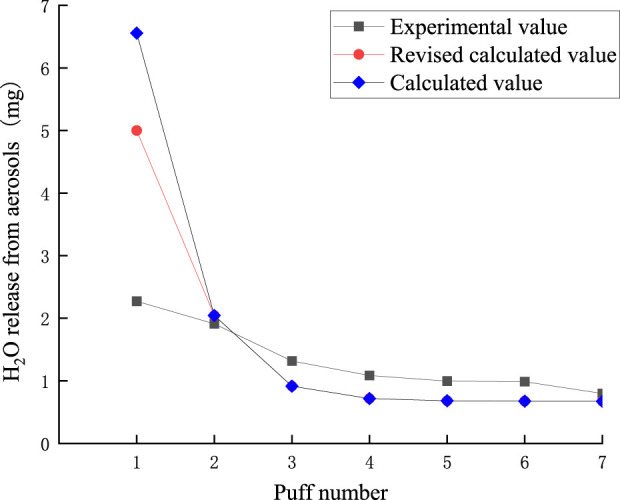
Comparison of calculated and experimental values of moisture release in aerosol.

The puff-by-puff release of moisture from the aerosol can be calculated by summarizing the data from the puff-by-puff changes in each functional segment. The comparison between the calculated and experimental values of moisture release in the aerosol is shown in Figure 17. There is a significant deviation between the experimental and calculated values in the first puff. Moisture was not captured in its entirety when released in the first puff, and there was a large loss of mass. This may be Due to the lower boiling point of moisture after preheating the smoker, and some of the water escapes into the air or condenses directly on the inner wall of the smoker after reaching the boiling point quickly. Meanwhile, from the previous discussion, it can be seen that the loss of other key components is also high in the first puff, which may be due to the fact that the other key components are also affected by the moisture at this time, and the synergistic escape leads to the loss of mass. Referring to Figure 1 in 3.2, it can be seen that the actual transfer efficiency of moisture per orifice is close to 0.8. Therefore, in order to more accurately predict the amount of moisture released in the aerosol, a correction factor λ = 0.8 was introduced into the model. As shown in Figure 14, the RMSE value between the predicted results and the measured moisture values was 1.06, the MAE value is 0.62. the deviation of the first puff after correction was reduced to approximately 3 mg, while the deviations for the other puffs were smaller. This suggests that the accuracy of predicting the puff-by-puff release of moisture from the second to the seventh puff is higher.

Moisture puff-by-puff release:

$$q_{w}\left(n\right)=\lambda_{w,1}\left(n\right)\Delta m_{w,1}\left(n\right)+\Delta m_{w,2}\left(n\right)+\Delta m_{w,3}\left(n\right)+\Delta m_{w,4}\left(n\right)$$ (28)

Based on the results of the study, we can observe that the retention of key components in different cigarette segments as well as the residual amounts in tobacco segments showed significant regular changes. Among them, the residual amounts of propylene glycol and nicotine showed similar trends in all segments, with an overall exponential decrease, whereas the residual amount of glycerol decreased in a linear manner, and the residual amount of moisture gradually stabilized after a sharp decrease in the first puff. It is worth noting that the large loss of water is also accompanied by the loss of other components; therefore, the release of water and the amount of water added need to be strictly controlled during product design. Meanwhile, the release pattern of the components in the tobacco section determines their release pattern in aerosol, so the stable release of the components in the tobacco section needs to be controlled in order to improve the stability of the release in the aerosol.

The factors affecting the retention effect may be related to the boiling point of the retained substances, especially in the hollow section and the cooling section, we observed the best retention effect. It is noteworthy that in the hollow section, glycerol not only played a good retention role but also realized the release of propylene glycol and water through its unsaturated state, which echoed the characteristic of water not only being retained but also released in the cooling section.

These findings reveal a close relationship between the retention characteristics of key components in the filament section and the design of cigarettes, and provide a theoretical basis for further optimization of tobacco products.

## 4 Conclusion

In this study, puff-by-puff analytical models were proposed to analyze the retention of key components in each segment of the NSC as well as the residual amounts in the tobacco segment. The results showed that the residual amounts of propylene glycol and nicotine in the tobacco section generally decreased exponentially, while the residual amount of glycerol decreased linearly; the moisture decreased sharply after the first sip and then leveled off. It is worth noting that the loss of water in the first puff after preheating can be substantial and is accompanied by the loss of other components, so the release and addition of water need to be strictly controlled in the design of tobacco products. In addition, the release pattern of the components in the tobacco section directly affects their performance in aerosol, so in order to improve the release stability of the product, it is necessary to strictly control the release of the tobacco section. The factors affecting the retention effect may be related to the boiling point of the material and the retained substance, and the best retention effect was observed in the hollow section and the cooling section. In particular, in the hollow section, glycerol not only played a good retention role but also promoted the release of propylene glycol and water through the unsaturated state, which echoed the characteristics of water that could be both retained and released in the cooling section. These findings reveal the close connection between the retention of key components and the design of aerosol and provide an important theoretical basis for optimizing the improvement of aerosol release stability.

## Data Availability

The original contributions presented in the study are included in the article/supplementary material, further inquiries can be directed to the corresponding authors.
